# Publicly versus privately funded cardiac rehabilitation: access and adherence barriers. A cross-sectional study

**DOI:** 10.1590/1516-3180.2020.0782.R1.31052021

**Published:** 2022-01-05

**Authors:** Giovanna Lombardi Bonini Borges, Mayara Moura Alves da Cruz, Ana Laura Ricci-Vitor, Paula Fernanda da Silva, Sherry Lynn Grace, Luiz Carlos Marques Vanderlei

**Affiliations:** I PT. Physiotherapist, Department of Physiotherapy, School of Technology and Sciences, Universidade Estadual Paulista (UNESP), Presidente Prudente (SP), Brazil.; II PT, MSc. Physiotherapist and Doctoral Student, Department of Physiotherapy, School of Technology and Sciences, Universidade Estadual Paulista (UNESP), Presidente Prudente (SP), Brazil.; III PT, PhD. Professor, Escola Superior de Saúde Egas Moniz (ESSEM), Egas Moniz - Cooperativa de Ensino Superior (CRL), Almada, Setúbal, Portugal.; IV PT. Physiotherapist and Master's Student, Department of Physiotherapy, School of Technology and Sciences, Universidade Estadual Paulista (UNESP), Presidente Prudente (SP), Brazil.; V PhD, CRFC. Professor, Faculty of Health, York University, Toronto, Ontario, Canada; and Senior Scientist, KITE & Peter Munk Cardiac Centre, University Health Network, University of Toronto, Toronto, Ontario, Canada.; VI PhD. Professor, Department of Physiotherapy, School of Technology and Sciences, Universidade Estadual Paulista (UNESP), Presidente Prudente (SP), Brazil.

**Keywords:** Cardiac rehabilitation, Cardiovascular diseases, Delivery of health care, Private sector, Surveys and questionnaires, Public sector, Barriers to cardiac rehabilitation, Public healthcare system, Patients’ characteristics

## Abstract

**BACKGROUND:**

Cardiac rehabilitation (CR) barriers are well-understood in high-resource settings. However, they are under-studied in low-resource settings, where access is even poorer and the context is significantly different, including two-tiered healthcare systems and greater socioeconomic challenges.

**OBJECTIVE:**

To investigate differences in characteristics of patients attending publicly versus privately funded CR and their barriers to adherence.

**DESIGN AND SETTING:**

Observational, cross-sectional study in public and private CR programs offered in Brazil.

**METHODS:**

Patients who had been attending CR for ≥ 3 months were recruited from one publicly and one privately funded CR program. They completed assessments regarding sociodemographic and clinical characteristics and the CR Barriers Scale.

**RESULTS:**

From the public program, 74 patients were recruited, and from the private, 100. Participants in the public program had significantly lower educational attainment (P < 0.001) and lower socioeconomic status (P < 0.001). Participants in the private program had more cognitive impairment (P = 0.015), and in the public program more anxiety (P = 0.001) and depressive symptoms (P = 0.008) than their counterparts. Total barriers among public CR participants were significantly higher than those among private CR participants (1.34 ± 0.26 versus 1.23 ± 0.15/5]; P = 0.003), as were scores on 3 out of 5 subscales, namely: comorbidities/functional status (P = 0.027), perceived need (P < 0.001) and access (P = 0.012).

**CONCLUSION:**

Publicly funded programs need to be tailored to meet their patients’ requirements, through consideration of educational and psychosocial matters, and be amenable to mitigation of patient barriers relating to presence of comorbidities and poorer health status.

## INTRODUCTION

Cardiovascular rehabilitation (CR) programs are recommended in clinical guidelines,^1,2^ because participation results in significantly lower mortality and morbidity,^3^ including in low and middle-income countries (LMICs).^4^ However, CR participation remains low, at around 20%-30% in high-income countries,^5–7^ and 14% in LMICs such as Brazil.^8^

The reasons for underuse of CR have been well-characterized in high-resource settings^7,9,10^ and include factors at the healthcare system, provider and patient levels. However, barriers in lower-resource settings have not been well-studied. A recent review identified only 13 studies globally,^11^ and there are also few studies in South America^12^ or Brazil to date.^13–16^ This is problematic, given the different contexts in these settings. Firstly, patients would be more socioeconomically disadvantaged, and hence face different barriers. Secondly, healthcare systems are more often two-tier.^17^ So, for example, half of CR programs in Brazil are solely publicly-funded (53.3%), a third privately-funded, and the remainder a mixture.^18^ It has been established that CR funding sources affect program characteristics, such as scale, healthcare providers on the team and component comprehensiveness.^17^ However, to our knowledge, it has yet to be investigated how barriers might differ for patients accessing privately and publicly-funded programs in any country worldwide.^19^

## OBJECTIVES

Therefore, the objectives of this study were to compare: (1) the sociodemographic and clinical characteristics of patients accessing publicly versus privately funded CR programs; and (2) multi-level barriers to adherence in each of these types of programs. While the world needs more CR,^20^ and offering privately funded programs may enable greater availability, the CR community needs to consider the inequities that this might raise.

## METHODS

### Design and procedure

This was an observational cross-sectional study. Participants signed an informed consent statement. The local ethics committee approved all procedures on June 28, 2018 (CAAE number: 88504718.0.0000.5402).

A convenience sample was recruited between March and August 2019. Participants in the public or private CR programs offered in the city of Presidente Prudente, São Paulo, Brazil, were approached with a view to inviting them to take part in this study and undergo assessments. These assessments were administered by physiotherapists who were not part of the programs.

### Setting

The publicly funded CR program for this study is offered by the Cardiology Division of the Center for Studies and Attendance in Physiotherapy and Rehabilitation, School of Technology and Sciences, Universidade Estadual Paulista (UNESP), Presidente Prudente, São Paulo, Brazil. The CR program is funded by the Brazilian National Health System and is delivered through the physiotherapy program. The program is indefinite in length (i.e. phase II and maintenance).

The privately funded CR program is offered through the city's Heart Institute. The program is funded by the patient or through medical health insurance (25.9% of Brazilians have health insurance).^21^ Most patients who use the private program have a health plan, for which they pay a monthly fee. This health plan covers 36 sessions, after which it is necessary to request coverage of further sessions if the doctor perceives more are required. When the patient does not have a health plan, they pay out-of-pocket monthly (R$ 390.00).

To start either program, patients require a written medical referral. The public program offers sessions three times/week, while the private program offers two to three per week, depending on the patient. In both programs, exercises are performed in groups; the public program serves on average 18 patients/session and the private one, 12 patients/session. With regard to staffing, in the private program, care is delivered by physiotherapy cardiology specialists; in the public program, care is provided by physiotherapy students supervised by professors.

The programs are primarily centered on structured exercises, and the exercise prescriptions are quite consistent between programs: they are based on heart rate reserve, and are re-evaluated each month. Exercises in the public program are done on treadmills and stationary bikes. In the private program, there are also resistance exercises. In addition, in the public program, there are group educational lectures and patients are provided with written materials. In the private program, there is informal counselling regarding risk factor control during the one-to-one sessions only.

### Participants

The inclusion criteria were that the participants needed to be aged over 18 years, with a diagnosis of cardiovascular disease or with cardiovascular risk factors (as per the program inclusion criteria), and needed to have been in the CR program for ≥ 3 months (frequency of attendance was not considered). There were no exclusion criteria.

### Measurements

The independent variable of interest was CR program funding type (public or private), which was coded based on the program attended. For objective one, the participants’ sociodemographic characteristics (e.g. age, sex, education and work status) and clinical characteristics (e.g. body mass index, CR indication/cardiac diagnosis and number of months in CR) were first assessed. The participants then completed psychometrically-validated scales assessing factors that are known to impact CR access and which may be particularly important in lower-resource settings, along with the CR Barriers Scale (CRBS; https://sgrace.info.yorku.ca/cr-barriers-scale/crbs-instructions-and-languages-translations/).

To quantify the participants’ socioeconomic level, a questionnaire from the Brazilian Association of Market Research Companies (ABEP) was administered. This asks about education level, family income, possession of certain items (e.g. number of televisions) and services offered in patients’ homes.^22^

To evaluate cognitive function, the psychometrically validated Brazilian-Portuguese version of the Mini-Mental State Examination (MMSE)^23^ was used. The test scores were adjusted based on level of education^24^ and categorized based on the presence of any cognitive impairment (e.g. participants who had four years of education and scored less than 25 were considered at least mildly cognitively impaired).

To quantify mental health symptoms, the psychometrically validated Brazilian-Portuguese version of the Hospital Anxiety and Depression Scale (HADS)^25^ was administered.

Lastly, CR barriers were assessed in relation to the second objective. The psychometrically validated Brazilian-Portuguese version of the Cardiac Rehabilitation Barriers Scale (CRBS) was administered.^13^ This assesses patient perceptions of 21 barriers at the healthcare system, healthcare provider and patient levels on a scale from 1 (“strongly disagree”) to 5 (“strongly agree”). Higher scores indicate higher barriers to CR adherence.^26^ A total mean score is computed, and there are five subscales: comorbidities/functional status, perceived need, personal/family issues, travel/work conflicts and access.

### Statistical analysis

To investigate differences in patient characteristics and barriers between participants attending public versus private programs, Fisher's exact tests or independent-sample t tests were used (or the Mann-Whitney U test if the variables were not normally distributed, as per the Kolmogorov-Smirnov test), as appropriate. Statistical significance was set at 5%. The analyses were performed using the IBM Statistical Package for the Social Sciences (SPSS) software, version 22.0 (SPSS Inc., Chicago, Illinois, United States).

## RESULTS

During the period of this study, 178 patients were approached, of whom 174 (97.75%) participated; 57.5% were from the private program. The sample characteristics are shown in Table 1.

**Table 1:** Sociodemographic and clinical characteristics of the participants, according to cardiac rehabilitation program funding type

Characteristics		Type of CR program		P
		Public	Private	
		(n = 74)	(n = 100)	
**Sociodemographic**				
**Age (years)**		65.61 ± 11.01	65.24 ± 14.22	0.315
**Sex (male)**		43 (58.11%)	65 (65.00%)	0.430
**City (same as CR location)**		68 (91.89%)	93 (93.00%)	0.779
**Work status (working)**		20 (27.03%)	40 (40.00%)	0.079
**Highest education level**				
	Completed high school	32 (43.24%)	29 (29.00%)	< 0.001
	Completed more than high school	42 (56.76%)	71 (71.00%)	
**Socioeconomic level** *				
	A	15 (20.27%)	55 (55.00%)	< 0.001
	B1	17 (22.97%)	21 (21.00%)	
	B2	27 (36.49%)	19 (19.00%)	
	C1	11 (14.86%)	2 (2.00%)	
	C2	4 (5.40%)	3 (3.00%)	
	D or E	0	0	
**Clinical**				
**Duration of CR (months)**		**77.38 ± 76.98**	**29.78 ± 21.68**	**< 0.001**
**Diagnoses/indications for CR**				
	CVD	62 (83.78%)	90 (90.00%)	0.253
	Ischemic heart disease	42 (67.74%)	68 (75.55%)	0.153
	Heart failure	13 (20.97%)	8 (8.89%)	0.063
	Valve diseases	2 (3.23%)	7 (7.78%)	0.304
	Rhythm disorders	2 (3.23%)	6 (6.67%)	0.469
	Other	3 (4.05%)	1 (1.00%)	0.041
**Risk factors**		**12 (16.22%)**	**10 (10.00%)**	**0.253**
	Arterial hypertension	11 (91.67%)	9 (90.00%)	0.241
	Family history	1 (8.33%)	1 (10.00%)	1.000
	BMI (kg/m²)	29.16 ± 4.71	28.81 ± 4.57	0.592
**Cognitive impairment (MMSE)**				
	Subthreshold	45 (60.81%)	48 (48.00%)	0.015
	At least mild	29 (39.19%)	52 (52.00%)	
	Mean ± SD	26.54 ± 3.00	27.66 ± 2.09	
**HADS - anxiety**				
	Unlikely anxiety	59 (79.73%)	89 (89.00%)	0.001
	Possible anxiety	12 (16.22%)	8 (8.00%)	
	Probable anxiety	3 (4.05%)	3 (3.00%)	
	Mean ± SD†	5.00 ± 3.38	3.43 ± 3.43	
**HADS - depressive symptoms**				
	Unlikely depression	64 (86.49%)	94 (94.00%)	0.008
	Possible depression	8 (10.81%)	4 (4.00%)	
	Probable depression	2 (2.70%)	2 (2.00%)	
	Mean ± SD†	4.05 ± 3.03	2.93 ± 2.62	

Note: The results are expressed as percentages and absolute numbers or as means and standard deviations (e.g. age and BMI).

* For socioeconomic level, A: 45-100 points; B1: 38-44 points; B2: 29-37 points; C1: 23-28 points; C2: 17-22 points; and D and E: 0-16 points.

† Scores ranged from 0 to 7, and higher scores indicated greater symptom burden.

MMSE = Mini-Mental State Examination; HADS = Hospital Anxiety and Depression Scale; BMI: body mass index; CVD = cardiovascular diseases; CR = cardiac rehabilitation; kg = kilograms; m = meters.

*Differences tested using t tests (or Mann-Whitney U test when data were not normally distributed) or Fisher's exact tests, as applicable.

With regard to sociodemographic characteristics, the participants in the public program had significantly lower educational attainment and lower socioeconomic status (plus a trend regarding work status). With regard to clinical characteristics, the participants in the private program had more cognitive impairment, and in the public program more anxiety and depressive symptoms than their counterparts. Participants were in the public program for significantly longer durations than those in the private program. Moreover, the total barriers were higher, the longer the participants were in the program (r = 0.244; P < 0.05).

As shown in Table 2, the total barrier scores in this sample of participants attending CR for ≥ 3 months were quite low. Regardless of the program accessed, travel/work conflicts were the greatest barrier, followed by personal/family issues and comorbidities/functional status. There was an open-ended question about any other barriers; no unique barriers were raised by participants.

**Table 2:** Cardiac rehabilitation barriers according to program funding type

Barriers	Public (n = 74)	Private (n = 100)	Total (n = 174)	P
10…travel	2.77 ± 1.94	2.63 ± 1.94	2.69 ± 1.93	0.600
14…other health problems prevent me from going	2.56 ± 1.95	2.20 ± 1.81	2.35 ± 1.88	0.168
4…of family responsibilities	1.93 ± 1.63	1.69 ± 1.49	1.79 ± 1.55	0.290
12…of work responsibilities	1.83 ± 1.61	1.71 ± 1.53	1.76 ± 1.56	0.475
8…severe weather	1.67 ± 1.70	1.21 ± 0.84	1.41 ± 1.30	0.029
11…of time constraints	1.44 ± 1.21	1.07 ± 0.50	1.23 ± 0.89	0.003
3…of transportation problems	1.29 ± 0.88	1.11 ± 0.63	1.19 ± 0.75	0.012
13…I don't have the energy	1.12 ± 0.75	1.06 ± 0.42	1.11 ± 0.59	0.120
1…of distance	1.23 ± 0.76	1.00 ± 0.00	1.10 ± 0.51	< 0.001
2…of cost	1.07 ± 0.25	1.04 ± 0.40	1.05 ± 0.34	0.045
20…it took too long to get referred and into the program	1.00 ± 0.00	1.06 ± 0.42	1.03 ± 0.32	0.214
15…I am too old	1.00 ± 0.00	1.04 ± 0.40	1.02 ± 0.30	0.386
9…I find exercise tiring or painful	1.04 ± 0.26	1.01 ± 0.00	1.02 ± 0.17	0.101
6…I don't need cardiac rehab	1.03 ± 0.16	1.00 ± 0.00	1.01 ± 0.11	0.101
7…I already exercise at home, or in my community	1.03 ± 0.16	1.00 ± 0.00	1.01 ± 0.11	0.101
5…I didn't know about cardiac rehab	1.01 ± 0.12	1.00 ± 0.00	1.01 ± 0.08	0.248
21…I prefer to take care of my health alone, not in a group	1.01 ± 0.12	1.00 ± 0.00	1.01 ± 0.08	0.248
16…my doctor did not feel it was necessary	1.00 ± 0.00	1.00 ± 0.00	1.00 ± 0.00	0.999
17… many people with heart problems don't go, and they are fine	1.00 ± 0.00	1.00 ± 0.00	1.00 ± 0.00	0.999
18… I can manage my heart problem on my own	1.00 ± 0.00	1.00 ± 0.00	1.00 ± 0.00	0.999
19… I think I was referred, but the rehab program didn't contact me	1.00 ± 0.00	1.00 ± 0.00	1.00 ± 0.00	0.999
**Total mean barrier**	**1.34 ± 0.26**	**1.23 ± 0.15**	**1.28 ± 0.21**	**0.003**
F1- Comorbidities/functional status	1.33 ± 0.43	1.21 ± 0.30	1.28 ± 0.37	0.027
F2- Perceived need	1.15 ± 0.29	1.03 ± 0.16	1.09 ± 0.23	< 0.001
F3- Personal/family issues	1.34 ± 0.56	1.23 ± 0.50	1.28 ± 0.53	0.131
F4- Travel/work conflicts	2.29 ± 1.40	2.17 ± 1.29	2.22 ± 1.33	0.630
F4- Access	1.06 ± 0.18	1.02 ± 0.14	1.04 ± 0.16	0.012

Mann-Whitney U test for differences between groups; F = factors/subscales.

In the “Barriers” column, the questions are presented in order from highest to lowest average score, as the question number and the summarized wording of the question.

As also shown in Table 2, the barriers were significantly higher among participants accessing the public program than among those accessing the private program. Moreover, scores on three of the five subscales were significantly higher among participants accessing the public program than among those accessing the privately funded program.

## DISCUSSION

There have been few studies on CR barriers outside of Western, high-income settings.^11^ In many of these countries, the healthcare systems are two-tier. It is known that there may be differences in program quality, and that there are significant differences in cost according to funding source,^17^ yet to our knowledge there has been no investigation of how this impacts patients. In this study, we began to investigate differences in the nature of patients accessing these programs, and how their barriers to adherence might differ, and indeed some important differences emerged.

It was promising to observe fewer differences than expected, in the characteristics of those accessing a publicly funded program rather than a privately funded program. For instance, there were no differences with regard to sex, age or diagnostic indication. As expected, the chief differences were socioeconomic, which are likely to explain the differences in mental health as well as cognition.^27^

The differences in the nature of patients accessing public or private programs, if replicated, hold implications for program delivery. Public programs would need to consider the health literacy of their patients, and tailor their educational programming accordingly.^28^ They would also want to ensure that they have staff who can assess and treat mental health issues, or have a close relationship with a referral source that does not have a long waitlist. Private programs could serve as important settings where patients who need more staff time could safely receive CR. If so, staff would need to have specialized training to successfully work with these patient groups.

The top barriers observed here were consistent with those reported in other studies, in Brazil, South America and beyond.^12,14,29–32^ Overall, the barriers were low, which was consistent with other CRBS studies in enrollees.^33^ This was to be expected, given the sample was composed of patients who had already completed ≥ 3 months of CR. Still, the patients accessing public programs did report significantly more barriers to adherence than did their counterparts in private programs. Socioeconomic differences in the cohorts do seem to explain the differences; for example, factors such as transportation costs, distance, time constraints and not getting support from healthcare providers to attend were more strongly endorsed by patients in the public than in the private system. Efforts to tackle the social determinants of health continue to be needed.

### Study limitations

Caution is warranted when interpreting these results. Their generalizability is limited, particularly given that we sampled from only one public and one private program. Moreover, the programs were of long duration, compared with other programs internationally.^34^ This study was also limited to participants who had been able to access CR and had adhered to the program for ≥ 3 months. Arguably these participants were among the few who had been able to successfully access and adhere to CR, even in a low-resource setting. In future studies, the barriers among participants should be investigated at the time of diagnosis (considering that referral is perceived as the main barrier in Brazil),^18^ as well as very early in their program. Lastly, the sample size was modest, and this was the first study examining these differences. Therefore, replication is warranted prior to implementing any changes based on these preliminary findings.

## CONCLUSIONS

In summary, as expected, but for the first time, we have shown that within a two-tier healthcare system in a lower-resource setting, patients accessing publicly funded CR programs are of significantly lower socioeconomic status and have poorer mental health and cognitive ability than those accessing privately funded programs. Publicly funded programs will need to tailor their delivery to meet the needs of their patients through educational and psychosocial programming. While referral and time conflicts remain key barriers in these settings, once patients do access CR, the barriers are greater for those in publicly funded programs than in privately funded ones, particularly with regard to comorbidities/functional status, perceived need and access.
